# Trans-Resveratrol Oral Bioavailability in Humans Using LipiSperse™ Dispersion Technology

**DOI:** 10.3390/pharmaceutics12121190

**Published:** 2020-12-08

**Authors:** David Briskey, Amanda Rao

**Affiliations:** 1 School of Human Movement and Nutrition Sciences, University of Queensland, Brisbane, QLD 4067, Australia; D.Briskey@uq.edu.au; 2 RDC Clinical, Newstead, Brisbane, QLD 4006, Australia

**Keywords:** resveratrol, LipiSperse^®^, absorption, bioavailability, pharmacokinetics

## Abstract

Resveratrol is a naturally produced compound that has been well researched for its potential health benefits. The primary hindrance towards resveratrol’s therapeutic efficacy is its traditionally poor oral bioavailability. LipiSperse^®^ is a novel delivery system designed to increase the dispersion of lipophilic ingredients, like resveratrol, in aqueous environments. This single-dose, double-blind, randomized study compared the pharmacokinetics of a commercially available resveratrol with (Veri-Sperse^®^) and without (Veri-te) the LipiSperse^®^ delivery complex. Healthy adults randomly received a single dose of either 150 Veri-te, 75 Veri-Sperse^®^, or 150 mg Veri-Sperse^®^. Venous blood samples were taken prior to dosing in a fasted state and at 0.5, 1, 2, 3, 4, 5, 6, 8 and 24 h post supplementation. Plasma trans-resveratrol conjugates were measured by liquid-chromatography tandem mass spectrometry (LC-MS/MS). The area under the curve (*AUC*) (0–24 h), maximum concentration (*C_max_*), and time of maximum concentration (*T_max_*) of plasma conjugates were calculated. The 150 mg dose of Veri-Sperse^®^ had a 2-fold increase in absorption (*AUC*) and a 3-fold increase in *C_max_* of trans-resveratrol conjugates compared to 150 mg Veri-te. There was no statistical difference between 75 Veri-Sperse and 150 mg Veri-te for *AUC* or *C_max_* of resveratrol conjugates. These findings provide support for the use of LipiSperse^®^ to improve absorption of resveratrol.

## 1. Introduction

Resveratrol is a natural product produced in grapes and other plants as a defense against UV radiation, environmental stress, and fungal infections [1]. Due to its anti-inflammatory and vasoactive properties, resveratrol received early attention in traditional Chinese and Japanese medicine. Today, resveratrol accounts for more than 12,000 scientific studies and 170+ human clinical trials [2], making it a well-studied functional ingredient within the dietary supplement industry. Ingested resveratrol at dosages between 6 and 5000 mg per day have been documented as safe [3].

Originally known for its protective heart-health benefits [4], research indicates that resveratrol taken in modest amounts can play a broader role. Clinical studies administering 75 to 500 mg/day have shown that resveratrol can benefit: Vascular [5] and heart health [6], cognitive [7] and mental performance [8], bone mineralization [9], physical activity [10], positive modulation of the gut microbiome [11], eyesight [11], and oral inflammation [12]. Resveratrol’s efficacy is evidenced through the upregulation of enzymatic antioxidants (e.g., catalase (Cat), superoxide dismutase (SOD)), and markers for chronic inflammation (e.g., CRP, IL-6, TNF-α).

Resveratrol can be extracted from plants such as Japanese knotweed and grape seed, and can be chemically synthesized, or produced via fermentation. The origin can affect the ingredient’s purity and quality in terms of potential contaminants [13]. Despite not knowing resveratrol’s exact mechanism of action, it is thought to interact with multiple molecular targets associated with inflammation and aging. Research highlights that resveratrol follows a multifaceted approach and its beneficial effects are based on its molecular structure, as well as cellular and systemic functionality [14,15,16]. More specifically, resveratrol has been characterized to directly interact with over 20 cellular targets and on several cellular processes indirectly by modulating cellular signals [17,18].

Three fundamental mechanisms seem to be crucial for the observed health benefits. First, resveratrol has been characterized by activating the endothelial nitric oxide synthase (eNOS) and shown to increase NO production and bioavailability [19], which can facilitate vasodilation and blood circulation in the body and brain [20,21]. Second, resveratrol activates 5′ AMP-activated protein kinase (AMPK), which can modulate cellular energy and glucose uptake [22]. Lastly, resveratrol appears to influence the activity of proteins called sirtuins, specifically SIRT1. Sirtuins seem to be involved in modulating a number of cellular pathways often related to stress responses and the aging process [23,24,25].

Daily dosage levels are still under debate, but the majority of clinical studies have been conducted administering 150 mg/day of resveratrol [26]. Nevertheless, a study in thirty-six adults (40–80 years of age) were randomized to consume single doses of resveratrol (0, 75, 150, and 300 mg) and 75 mg showed the best efficacy for blood flow velocity measured during a cognitive battery of tests [20]. Wong et al. (2016) suggested a daily intake of 150 mg resveratrol but divided into two doses of 75 mg in the morning and 75 mg in the evening. The argument for this regimen is to maintain a modest amount of resveratrol continuously in the blood plasma [19,27].

Fundamentally, like other polyphenols, oral intake of resveratrol has a relatively low bioavailability since the compound is rapidly metabolized by the microbiota and in the liver by the first pass metabolism. Through this metabolism, resveratrol can be conjugated with sulfate and glucuronide groups, resulting in the three primary metabolites as resveratrol-3-*O*-sulfate, resveratrol-4′-*O*-glucuronide, and resveratrol-3-*O*-glucuronide [28].

Researchers investigated how resveratrol metabolites can provide an intracellular pool of free resveratrol, and the amount of total resveratrol (resveratrol plus resveratrol metabolites) should be considered when measuring the bioavailability [29]. In fact, radioactively labeled resveratrol presented an absorption of a 25 mg oral dose of resveratrol to be at least 70%, resulting in a maximum peak plasma concentration (*C_max_*) of resveratrol and metabolites of not more than 491 ± 90 ng/mL (about 2 µM) [27]. However, the rapid absorption and extensive metabolism are often reported as a potential limitation of resveratrol efficacy. To overcome the pharmacokinetics and improve bioavailability of orally ingested resveratrol commonly reported as <1% free resveratrol in blood plasma, several delivery techniques have been developed including micronization, which increases the surface area or nanoencapsulation in lipid nanocarriers or liposomes and micelles [30].

As previously described, LipiSperse^®^ is a novel delivery system designed to increase the dispersion of lipophilic agents in aqueous environments [31]. The addition of lipophilic active ingredients often leads to decreased active load in final formulations. LipiSperse^®^ is a mixture of surfactants, polar lipids, and solvents. LipiSperse^®^ increases the wettability of the crystal by lowering the surface tension, which allows it to disperse in water and prevent the crystals from agglomerating. Repulsive forces between the particles prevent agglomeration or aggregation: Allowing for particle dispersion. The present study aimed to compare the pharmacokinetics of a single dose of commercially available resveratrol (Veri-te) with a resveratrol-LipiSperse^®^ delivery complex (Veri-Sperse^®^. The results showed that Veri-Sperse^®^ significantly increased the plasma concentration of trans-resveratrol conjugates over 24 h when compared to an equivalent amount of Veri-te. This indicates that the LipiSperse^®^ delivery system improves the oral bioavailability of resveratrol.

## 2. Materials and Methods

### 2.1. Study Design

A single-dose, double-blind, randomized study was used to evaluate the absorption of resveratrol with and without LipiSperse^®^. This study was registered with the Australian New Zealand Clinical Trials Registry (ACTRN12619000953134) and approved by the University of Queensland Human Research Ethics Committee (20/12/2018; HREC 2018002290) and conformed to the 1964 Declaration of Helsinki and its later amendments. Participants were healthy men and women, aged over 18 with inclusion criteria including normal dietary habits, body mass index between 18.5 to 35 kg/m^2^, and no history or evidence of clinically significant medical conditions. Exclusion criteria included any uncontrolled or serious illness, use of anticoagulant drugs, malignancy or treatment for malignancy within the previous two years, smoking, non-stable diet, chronic past or current alcohol abuse, allergy to any study ingredients, any diagnosed neurological conditions, use of supplements containing resveratrol, and participation in any other clinical trial in the past month. All participants provided written informed consent prior to participation.

Resveratrol absorption was measured from venous blood samples taken prior to dosing (*t* = 0) and at intervals of 0.5, 1, 2, 3, 4, 5, 6, 8, and 24 h post supplementation. Samples were obtained from a vein in the antecubital fossa using a cannula (BD, New Jersey) and 6 mL vacutainer containing Ethylenediaminetetraacetic acid (EDTA) (BD, New Jersey). Collected samples were immediately centrifuged at 4 °C for 10 min (2300 RPM) with aliquots of plasma stored at −80 °C.

Following a fasting blood sample, participants consumed a single dose of either:

- Group A (*n* = 12)—150 mg of Veri-te resveratrol;
- Group B (*n* = 12)—75 mg of Veri-te resveratrol with LipiSperse^®^ dispersion technology (Veri-Sperse^®^);
- Group C (*n* = 13)—150 mg of Veri-te resveratrol with LipiSperse^®^ dispersion technology (Veri-Sperse^®^).

All supplements were consumed with 250 mL of water.

### 2.2. Participants and Diet

Thirty-nine healthy male (*n* = 20) and female (*n* = 19) volunteers were recruited to take part in this study. Participants were required to avoid foods containing resveratrol from 48 h prior to testing until the collection of the final blood sample. During the first 8 h of testing, participants remained at the clinic and were provided with standardized meals (breakfast and lunch). Following the fasting blood sample and consumption of the capsule, participants were provided with a breakfast meal that contained no resveratrol and was low in fat (containing approximately 25.1 protein, 4.5 fat, and 92.7 g carbohydrates). Approximately four hours following the ingestion of the study supplement, a lunch was provided that was also a standardized meal containing no resveratrol. While at the clinic, each participant consumed approximately 50.8 protein, 17.6 fat, and 167.2 g of carbohydrate for an intake of 4521 kJ. Participants were monitored for adverse effects to the treatment for the duration of the trial. Thirty-seven participants completed the trial with 2 participants removed due to not being able to complete several consecutive blood draws.

### 2.3. Analysis of Trans-Resveratrol Conjugates

Plasma trans-resveratrol conjugates and derivatives such as trans-resveratrol-sulfate and trans-resveratrol-glucuronide were analyzed by liquid-chromatography tandem mass spectrometry (LC-MS/MS; Thermo Fisher Scientific, Waltham, MA, USA) using a previously published method [32]. For trans-resveratrol-sulfate and trans-resveratrol-glucuronide, plasma (100 µL) was transferred to a 2-mL microfuge tube containing 20 ng of an internal standard [trans-resveratrol-d4 (Cayman chemical #13130, Ann Arbor, MI, USA)]. To the tube, 1 mL of acetonitrile was added followed by vortex mixing and centrifugation (13,000 RPM for 10 min) to remove precipitated proteins. The resulting supernatant was transferred to a clean glass tube and dried under nitrogen at 37 °C. Once dry, the residue was reconstituted with 200 µL of 20% methanol/water, vortex mixed, sonicated, and transferred to an amber sample vial where 10 µL was injected for analysis. Freshly prepared trans-resveratrol standards with internal standard were analyzed along with samples each day. The concentrations of trans-resveratrol-sulfate and trans-resveratrol-glucuronide were calculated using analytical standards purchased from Sapphire Bioscience (Redfern, NSW, Australia; trans-resveratrol (item No. 70675), trans-resveratrol-3-sulfate (item No. R150040), and trans-resveratrol 3-*O*-beta-D-Glucuronide (item Uo. R150015) standards, and trans-resveratrol-d4 (item No. 13130) as the internal standard).

Chromatography was performed using a Synergi 4 μm Hydro-RP 80A (250 × 2.0 mm; Phenomenex, Torrance, CA, USA) maintained at 40 °C with a flow of 0.2 mL/min. Mobile phase A consisted of water with 0.2% formic acid, mobile phase B consisted of methanol with 0.2% formic acid, and mobile phase C consisted of isopropanol with 0.2% formic acid. The flow rate was set at 200 µL/min with the mobile phase starting with 88/10/2 % of mobile phase A, B, and C, respectively, and held for the 0.5 min. The phase was changed to 18/80/2 over 3 min and held for a further 8.5 min. The mobile phase was then returned to 88/10/2 over 1 min and held for 5 min for a total run time of 18 min.

A turbo ion spray interface was used and operated in negative ion mode. Acquisition was made in the multiple reaction monitoring mode using the following ions: Trans-resveratrol-d4 (231→189), trans-resveratrol-sulfate (307→227), and trans-resveratrol-glucuronide (403→227).

The assay was reproducible with an intra-assay precision of 4.1%, and inter-assay precision of 4.8%. The assay also showed good accuracy, with intra-assay concentrations within 91.6% and 103.7% of the expected value, and inter-assay concentrations within 88.6% to 105.1% of the expected value. The linear concentration range used was between 0.03 and 30 μg/mL with a lower limit of quantitation of 10 ng/mL when 100 μL of plasma was extracted.

### 2.4. Statistical Analysis

To account for the possibility of dietary resveratrol being present in the plasma, it was proposed that the absorption results be calculated as a change from baseline (baseline corrected) for each participant at each time-point. However, no free resveratrol or resveratrol conjugates were found in any baseline sample.

Measurements included: Area under the curve (*AUC*) (0–24 h calculated by the trapezoidal model), maximum concentration (*C_max_*), and time of maximum concentration (*T_max_*). Data was tested for normality using the D’Agostino–Pearson normality test. Subsequently, *t*-tests were performed to identify statistical differences between groups. All analysis was conducted using GraphPad Prism version 7.00 for Windows (GraphPad Software, La Jolla, CA, USA, www.graphpad.com).

### 2.5. Adverse Events

No adverse events to any of the treatments were reported. Two participants were excluded from the study due to an inability to provide blood after consent.

## 3. Results

The following results are a summary of the total trans-resveratrol-sulfate and trans-resveratrol-glucuronide analyses.

### Plasma Trans-Resveratrol Conjugate Analysis

**Figure 1:**
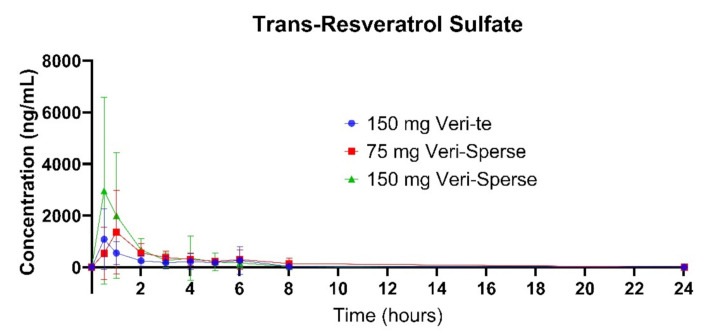
Plasma concentration of the trans-resveratrol sulfate over 24 h after a single dose.

**Figure 2:**
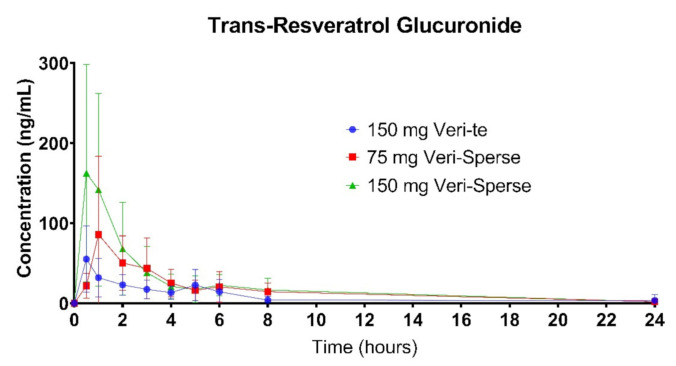
Plasma concentration of the trans-resveratrol glucuronide conjugates over 24 h after a single dose.

**Figure 3:**
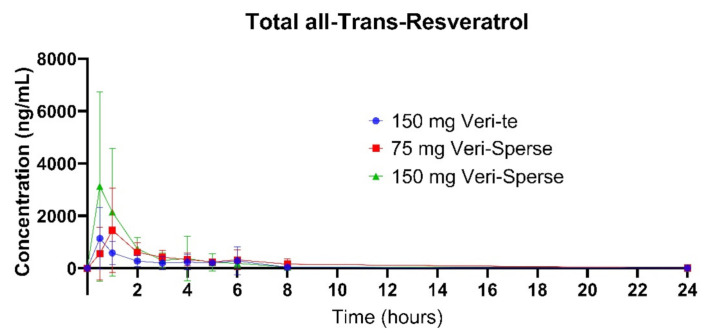
Plasma concentration for the sum of sulfate and glucuronide conjugates (all-trans-resveratrol) over 24 h after a single dose.

The 150 mg Veri-Sperse absorbed approximately 2 times more than the standard Veri-te 150 mg resveratrol with a *C_max_* of approximately 3 times that of the standard Veri-te resveratrol (Figure 1, Figure 2 and Figure 3). The differences in total absorption and *C_max_* were statistically significant between groups (Table 1 and Table 2; *p* < 0.05).

There was no statistically significant difference between the 75 Veri-Sperse and the 150 mg standard Veri-te resveratrol for the total area under the curve (*AUC*) or *C_max_*, indicating no significant difference between the two groups (Table 1 and Table 2).

## 4. Discussion

Current evidence supports the beneficial effects of resveratrol for a wide variety of health indications [4,5,6,7,8,9,10,11,12]. However, absorption may limit the beneficial use of resveratrol. Numerous strategies have therefore been developed to increase the absorption of resveratrol including nanoparticles and self-nanoemulsifying drug delivery systems (SNEDDS) [33,34,35]. Given the variation in methodology and study design used to quantify the absorption of resveratrol (e.g., delivery technology and dose used), it is difficult to compare absorption efficacy across the literature. As such, no one strategy is yet to emerge superior for absorption of orally dosed resveratrol.

Our trial was conducted under standardized conditions with the aim of assessing the levels of resveratrol and conjugates both prior to, and during the investigation. Consistent with similar research, baseline concentrations of resveratrol (as free and conjugates) were below the limit of detection in all groups; thus, we can confidently state there were no significant between-group differences in plasma resveratrol prior to dosage.

The current study examined the effects of LipiSperse^®^, a novel delivery system that uses dispersion technology to enhance the absorption of hydrophobic agents, on the absorption of a commercially available resveratrol ingredient. By attaching itself to the surface of resveratrol particles, LipiSperse^®^ acts as a dispersing agent to lower the hydrophobicity of resveratrol. Given this, the enhanced bioavailability reported in this study is likely due to increased gastrointestinal absorption secondary to a reduction in intermolecular forces as discussed in the introduction.

One variable often overlooked when comparing absorption study effects is the composition of food consumed during the trial. In particular, the composition of the meal consumed immediately after the capsule is taken (i.e., a meal high in fat is known to increase intestinal absorption) [36,37,38]. In the present study, participants consumed the supplement (Veri-te resveratrol or Veri-Sperse™) on a fasted stomach followed by the consumption of a low-fat breakfast and lunch containing no resveratrol. This procedure minimized extrinsic factors that could exert influence on the absorption of the supplement via increased intestinal absorption.

The aim of the present study was to demonstrate that bioavailability of trans-resveratrol can be increased when combined with LipiSperse^®^. Other absorption studies have shown that supplementation with doses up to 500 mg of resveratrol resulted in low absorption of free trans-resveratrol (1.48 to 47.3 ng/mL) [3,39,40]. Studies supplementing participants with doses of resveratrol of 5000 mg have shown that free resveratrol can reach concentrations as high as 538.8 ng/mL and generally increases in a dose dependent manner [3,41]. Supplementing with resveratrol for 1 to 4 weeks has shown to have an accumulative effect, allowing smaller doses to increase free trans-resveratrol concentrations circulating in the blood [3,42,43]. This is critical, as a long-term dose of 5000 mg is unsustainable, with increased side effects reported for doses over 2500 mg [41,42,43]. In line with the above, this study supported the observations of these previous studies in that limited free trans-resveratrol enters circulation. This is likely due to the metabolism of the trans-resveratrol in the gastrointestinal tract and liver to the resveratrol conjugates [44,45].

There are several forms of resveratrol (e.g., trans-, cis-, and dihydro-). This study focused on the trans-resveratrol form. For each of the resveratrol forms, there are 2 major conjugates of sulfate and glucuronide groups. Within these conjugate groups, there can be a number of forms (e.g., trans-resveratrol-3-sulfate and trans-resveratrol-4-sulfate). These forms possess identical molecular mass making them difficult to analytically separate. Furthermore, trans-resveratrol-4-sulfate is difficult to manufacture due to its instability. Therefore, the data presented here is the total for sulfate and glucuronide conjugates of the stated forms.

Furthermore, preliminary testing found that the free resveratrol and dihydro-resveratrol forms represented <1% of the total resveratrol appearing in the body. Therefore, this study focused on the conjugates of trans-resveratrol and showed the primary conjugate was trans-resveratrol-sulfate. The trans-resveratrol-sulfate groups conjugate accounting for as much as 95% of the data presented with the trans-resveratrol-glucuronide conjugate, accounting for the remaining data.

Due to the rapid metabolism and comparably low levels of free trans-resveratrol, this study focused on the trans-resveratrol conjugates (sulfates and glucuronides) to assess the efficacy of LipiSperse^®^ for increasing absorption. One possible reason for the rapid conversion of resveratrol to its conjugate form is for stability. Once the conjugated form has entered the bloodstream and been delivered to the target tissue, it has been reported that the parent compound is able to be regenerate to provide the reported beneficial in vivo effects [46,47]. However, even in the absence of regeneration of the parent compound, it has also been reported that the metabolites have biological effects that contribute to the reported in vivo effects [46,48].

This study showed that compared with a 150 mg dose of Veri-te trans-resveratrol, a single 150 mg dose of trans-resveratrol with LipiSperse^®^ resulted in a 2-fold increase in plasma concentration over 24 h (measured as *AUC*) and a 3-fold increase in *C_max_* (Table 1 and Table 2 and Figure 1) of both the sulfate and glucuronide conjugates. When the Veri-te 150 mg dose of resveratrol was compared to the 75 mg Veri-Sperse dose, there was no statistical difference between the *C_max_* or *AUC* for the conjugates analyzed. This suggests that a 75 mg dose of resveratrol with LipiSperse^®^ is not significantly different to a 150 mg dose of Veri-te resveratrol.

Using the *AUC* calculation to estimate the fraction of the supplement absorbed further highlights the difference between the standard and LipiSperse^®^ groups. The 150 mg Veri-te group showed a theoretical absorption of 7.9 mg compared to the 75 and 150 mg Veri-Sperse groups of 12.3 and 15.7 mg, respectively. When converted to a percentage of the dose provided, this absorption equates to a 5.2%, 16.5%, and 10.5% absorption of the dose provided for the 150 Veri-te, 75 Veri-Sperse, and 150 mg Veri-Sperse groups, respectively. However, this interpretation has to be taken with a degree of caution, as the rate of clearance (excretion and cellular uptake) has not been measured. If, for example, the rate of clearance was approximately linear with the rate of appearance, the aforementioned values would theoretically double, giving 10.4%, 33.0%, and 21.0% absorption fractions. Therefore, it is difficult to know what the true percentage of the supplement absorbed was. Despite this, even with the use of a novel delivery system such as LipiSperse^®^, there still appears to be room for the absorption of resveratrol to be significantly improved.

Comparison of data using *AUC*, however, has limitations. Firstly, as discussed, the clearance rate cannot be accurately taken into consideration. Secondly, the ability to determine when a participant has returned to baseline concentrations is difficult. When ratios of the data are compared, there are differences in the ratio of improvements in the *AUC* vs. *C_max_* between groups. For example, when the two Veri-Sperse groups were compared, the 150 mg dose achieved a 1.26 times greater *AUC*, yet a 2.3 times greater *C_max_*. One explanation for this is a “long tail” effect. As we were unable to sample blood at a frequency that would provide an accurate time for when the participant returned to baseline concentration, the 24-h time point data is used as the end point. This means that the *AUC* calculated from the 8- to 24-h time point may skew the data by providing a higher than expected measure. A second explanation for this may be the rate of absorption or clearance. At the lower dose, the resveratrol may be cleared (taken up by the cells) from the plasma at a greater percentage rate than in the 150 mg dose. This could be due to a limitation in the rate at which resveratrol can be taken up by the cells. If one exceeds this rate, any additional supplementation absorbed may stay in the plasma waiting for excretion or cellular uptake. This may result in a short-term spike in the plasma concentration at the point where resveratrol uptake is maximized. Additionally, the 150 mg dose may then maintain a higher clearance rate vs. the 75 mg dose for longer, subsequently affecting the *AUC*.

The final factor in the observed differences in *AUC* vs. *C_max_* may come down to the participants themselves. As we used a diverse group of individuals, the data had a degree of variability. This variability may result from a number of factors, the most likely factor being the participants’ gastrointestinal tract (GIT). An individual’s microbiome contributes significantly to absorption, be it a food or supplement. Each individual’s microbiome can be as unique as their fingerprint, and the effect this has is unable to be determined in a study of this nature. The participants may also have differences in the rate of resveratrol clearance. Some participants may have a faster cellular uptake rate than others. This may result in that person presenting a lower *AUC* and possibly *C_max_* than someone with a slow cellular uptake.

When the *C_max_* results of each resveratrol conjugate in this study were compared to previous literature, there were mixed results. Firstly, it is difficult to directly compare results from other studies due to variations in trial product (source and dose), duration and timing of blood samples collected, and method of sample detection (i.e., UV vs. mass spectrometry). When we compared the highest dose investigated here (150 mg) with the 500 mg doses investigated by Brown (2011) or Sergides (2016), we observed that our data on trans-resveratrol sulfate conjugates achieved consistently higher concentrations (3666 ng/mL vs. 563–1516 ng/mL) despite the lower dose. However, when comparing the glucuronide conjugates data, we observed that there appeared to be far more variability between the data sets. Our 150 mg dose achieved a *C_max_* of 200 ng/mL vs. a reported *C_max_* of between 186 and 4084 ng/mL for a 500 mg dose. The exact reason for the variability could be due to a number of factors, the most probable being the in vivo conjugation. It may be that in our study, the supplement was more susceptible to sulfate conjugation and that in other studies, Sergides et al. (2016) for example, the trans-resveratrol may have been more susceptible to glucuronidation. This could be due to either the composition of the supplement itself, or the microbiome composition of the participants. As each study was conducted in a different part of the world, it is likely that the diet consumed by each study population as a whole is different. This is likely to result in a difference in the microbiome between study groups and therefore potentially change the conjugation of the supplement. The effect, if any, this difference in conjugation may have on health however is unknown.

One observation made in the current study is that there is a small secondary peak in each resveratrol conjugate concentration between 4–6 h post consumption. This peak is likely caused by hepatic-recirculation [49], and does not necessarily reflect additional absorption of the dosed product. However, as this second peak is very minor in comparison to the total *AUC*, it is not considered to be of significance in affecting the results presented.

## 5. Conclusions

In conclusion, we examined the effect of a novel dispersion agent (LipiSperse^®^) on resveratrol absorption. Our findings indicate that plasma concentrations of resveratrol conjugates can be significantly increased when combined with LipiSperse^®^. This study therefore shows LipiSperse^®^ can be used to improve oral absorption of resveratrol.

## Figures and Tables

**Table 1:** Trans-resveratrol-sulfate absorption data.

Outcome Measure	150 mg Veri-te trans-Resveratrol	75 mg Veri-Sperse	150 mg Veri-Sperse
*AUC* (ng/mL/day) ^1^	2900 ± 1962	4563 ± 2891	5765.8 ± 4154 *
*C_max_* (ng/mL) ^2^	1201 ± 919.9	1591.4 ± 1645.8	3666.2 ± 3814.4 *
*T_max_* (h) ^3^	1.3 ± 1.7	1.4 ± 0.7	0.8 ± 0.6

* *p* < 0.05 compared to 150 mg Veri-te group; ^1^ *AUC* = area under the curve, ^2^ *C_max_* = maximum concentration, ^3^ *T_max_* = time for maximum concentration.

**Table 2:** Trans-resveratrol-glucuronide absorption data.

Outcome Measure	150 mg Veri-te trans-Resveratrol	75 mg Veri-Sperse	150 mg Veri-Sperse
*AUC* (ng/mL/day) ^1^	245 ± 145	373 ± 222	531 ± 333 *
*C_max_* (ng/mL) ^2^	60.9 ± 37.2	108.7 ± 90.6	200.7 ± 153 *
*T_max_* (h)^3^	1.2 ± 1.4	1.5 ± 0.9	1.0 ± 0.7

* *p* < 0.05 compared to 150 mg Veri-te group; ^1^ *AUC* = area under the curve, ^2^ *C_max_* = maximum concentration, ^3^ *T_max_* = time for maximum concentration.
